# Supplementary material for: Antileishmanial and Antiplasmodial Activities of Secondary Metabolites from the Root of Antrocaryon klaineanum Pierre (Anacardiaceae)
Source: Molecules. 2023 Mar 17;28(6):2730. doi: 10.3390/molecules28062730 (PMC10059057; doi:10.3390/molecules28062730)
Supplement: Supplementary file 1 [file molecules-28-02730-s001.zip › molecules-2245337-supplementary.pdf]

SUPPLEMENTARY MATERIALS

Antileishmanial and Antiplasmodial Activities of Secondary Metabolites from the Root of *Antrocaryon klaineanum* Pierre (Anacardiaceae)

Gabrielle Ange Amang à Ngnoung [1,2], Lazare S. Sidjui [2,3], Peron B. Leutcha [1,4], Yves O. Nganso Ditchou [1,5,*], Lauve R. Y. Tchokouaha [6], Gaëtan Herbette [7], Beatrice Baghdikian [8], Theodora K. Kowa [2], Desire Soh [5,9], Raoul Kemzeu [10], Madan Poka [11], Patrick H. Demana [11], Xavier Siwe Noundou [11,*], Alembert T. Tchinda [2], Fabrice Fekam Boyom [10], Alain M. Lannang [4,12] and Barthélemy Nyassé [5]

1 Department of Chemistry, Faculty of Science, University of Maroua, Maroua P.O. Box 814, Cameroon
2 Laboratory of Phytochemistry, Centre for Research on Medicinal Plants and Traditional Medicine, Institute of Medical Research and Medicinal Plants Studies, Yaoundé P.O. Box 13033, Cameroon
3 Bioorganic and Medicinal Chemistry Laboratory, Department of Organic Chemistry, Faculty of Science, University of Yaoundé I, Yaoundé P.O. Box 812, Cameroon
4 Natural Product and Environmental Chemistry Group (NAPEC), Department of Chemistry, Higher Teachers' Training College, University of Maroua, Maroua P.O. Box 55, Cameroon
5 Laboratory of Medicinal Chemistry & Pharmacognosy, Department of Organic Chemistry, Faculty of Science, University of Yaoundé I, Yaoundé P.O. Box 812, Cameroon
6 Laboratory of Pharmacology and Drugs Discovery, IMPM, Yaoundé P.O. Box 13033, Cameroon
7 Aix-Marseille Univ, CNRS, Centrale Marseille, FSCM, Spectropole, Campus de St Jérôme-Service 511, 13397 Marseille, France
8 Aix Marseille Univ, CNRS 7263, IRD 237, Avignon Université, IMBE, 27 Blvd Jean Moulin, Service of Pharmacognosy, Faculty of Pharmacy, 13385 Marseille, France
9 Department of Chemistry, Higher Teacher Training College Bambili, The University of Bamenda, Bambili, Bamenda P.O. Box 39, Cameroon
10 Antimicrobial and Biocontrol Agents Unit, Laboratory for Phytobiochemistry and Medicinal Plants Studies, Department of Biochemistry, Faculty of Science, University of Yaounde 1, Yaounde P.O. Box 812, Cameroon
11 Department of Pharmaceutical Sciences, School of Pharmacy, Sefako Makgatho Health Sciences University, Pretoria 0204, South Africa
12 Department of Chemical Engineering, School of Chemical Engineering and Mineral Industries, University of Ngaoundere, Ngaoundere P.O. Box 454, Cameroon
* Correspondence: nganso_yves@yahoo.fr (Y.O.N.D.); xavier.siwenoundou@smu.ac.za (X.S.N.); Tel.: +237-6-9946-1194 (Y.O.N.D.); +27-12-521-5647 (X.S.N.)

Table of Contents

Figure S1 : UV spectrum of compound **1** .... **Error! Bookmark not defined.**

Figure S2: LR-ESI-MS (+) of compound **1**... **Error! Bookmark not defined.**

Figure S3: LR-ESI-MS (-) of compound **1** .... **Error! Bookmark not defined.**

Figure S4: HR-ESI-MS (+) of compound **1**.. **Error! Bookmark not defined.**

Figure S5: MS/MS spectrum of the $[M+Na]^+$ ion at *m/z* 866.7 (Ecollision = 65 eV) of compound **1** .. **Error! Bookmark not defined.**

Figure S6: IR spectrum (KBr) of compound **1**... **Error! Bookmark not defined.**

Figure S7: ^{1}H-NMR (Acetone-d_6, 600 MHz) spectrum of compound **1** ..... **Error! Bookmark not defined.**

Figure S8: ^{1}H-NMR (CD_3OD, 600 MHz) spectrum of compound **1**.......... **Error! Bookmark not defined.**

Figure S9: COSY (Acetone-d_6) full spectrum of compound **1**..................... **Error! Bookmark not defined.**

Figure S10: COSY (Acetone-d_6) expanded spectrum of compound **1**........ **Error! Bookmark not defined.**

Figure S11: HSQC (Acetone-d_6) spectrum of compound **1** .... **Error! Bookmark not defined.**

Figure S12: HSQC (CD_3OD) spectrum of compound **1** .... **Error! Bookmark not defined.**

Figure S13: HMBC (Acetone-d_6) spectrum of compound **1** .... **Error! Bookmark not defined.**

Figure S14: HMBC (CD_3OD) spectrum of compound **1** .... **Error! Bookmark not defined.**

Figure S15: ^{13}C-NMR (Acetone-d_6, 150 MHz) spectrum of compound **1**... **Error! Bookmark not defined.**

Figure S16: ^{13}C-NMR (CD_3OD, 150 MHz) spectrum of compound **1** .... **Error! Bookmark not defined.**

Figure S17: ^{1}H-NMR (DMSO-d_6, 600 MHz) spectrum of compound **2**...... **Error! Bookmark not defined.**

Figure S18: ^{13}C-NMR (DMSO-d_6, 150 MHz) spectrum of compound **2**..... **Error! Bookmark not defined.**

Figure S19: ^{1}H-NMR (CD_3OD, 600 MHz) spectrum of compound **3**......... **Error! Bookmark not defined.**

Figure S20: ^{13}C-NMR (CD_3OD, 150 MHz) spectrum of compound **3** .... **Error! Bookmark not defined.**

Figure S21: ^{1}H-NMR (DMSO-d_6, 600 MHz) spectrum of compound **4**...... **Error! Bookmark not defined.**

Figure S22: HMBC spectrum of compound **4**... **Error! Bookmark not defined.**

Figure S23: ^{1}H-NMR ($CDCl_3$, 600 MHz) spectrum of compound **5 & 6**.... **Error! Bookmark not defined.**

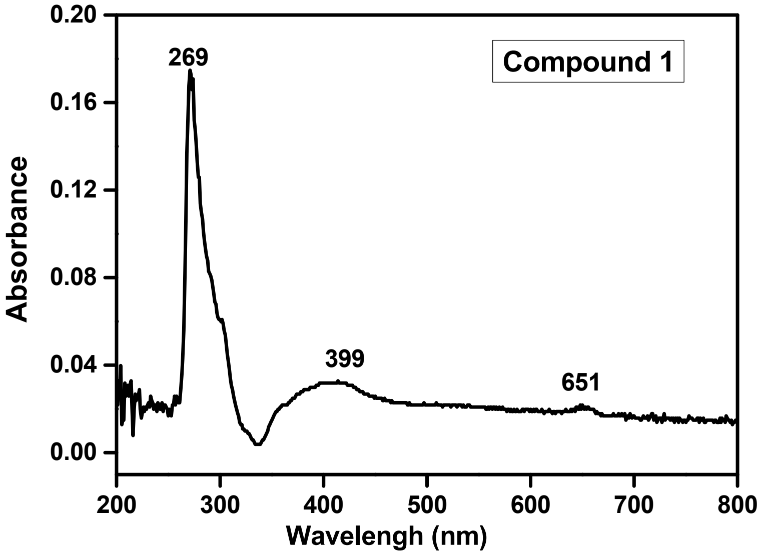

Figure S1 : UV spectrum of compound **1**

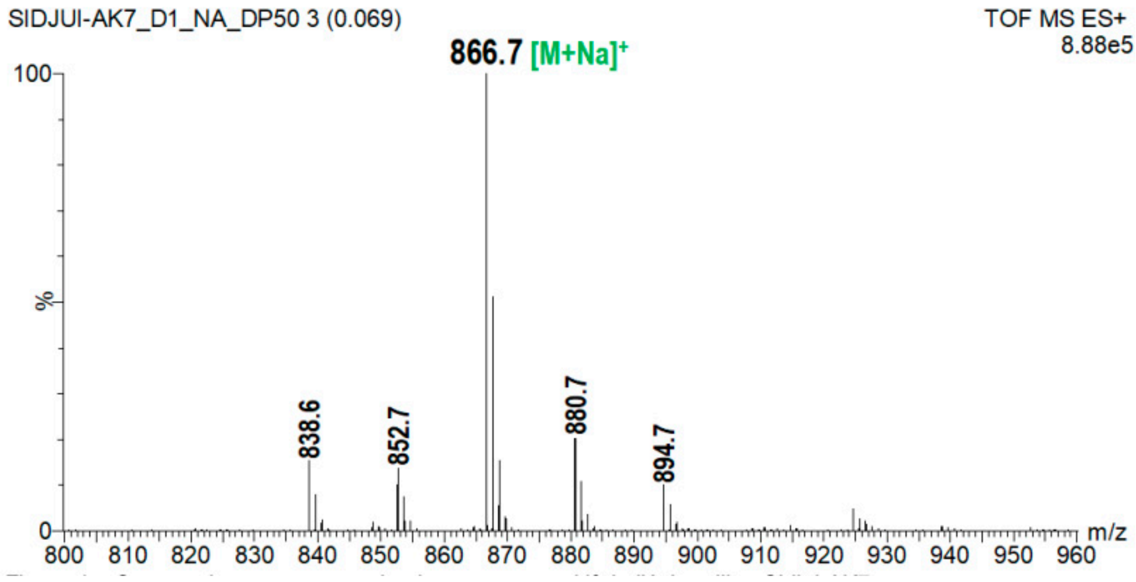

Figure S2: LR-ESI-MS (+) of compound **1**

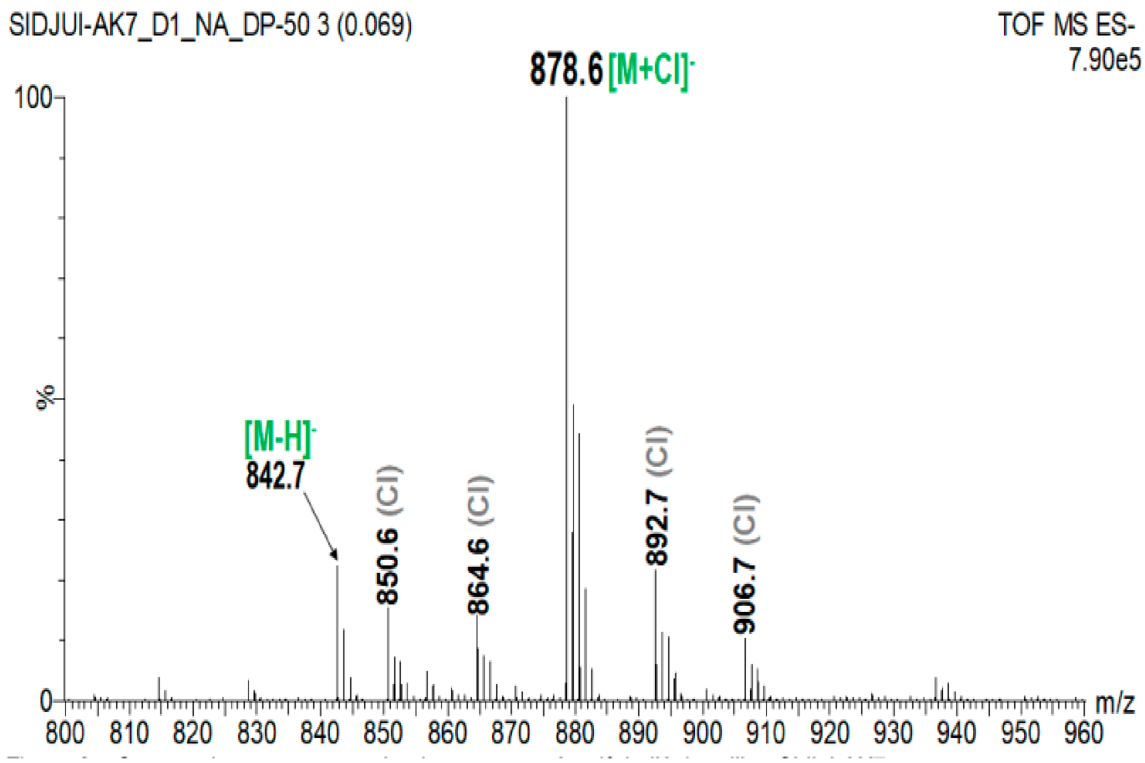

Figure S3: LR-ESI-MS (-) of compound **1**

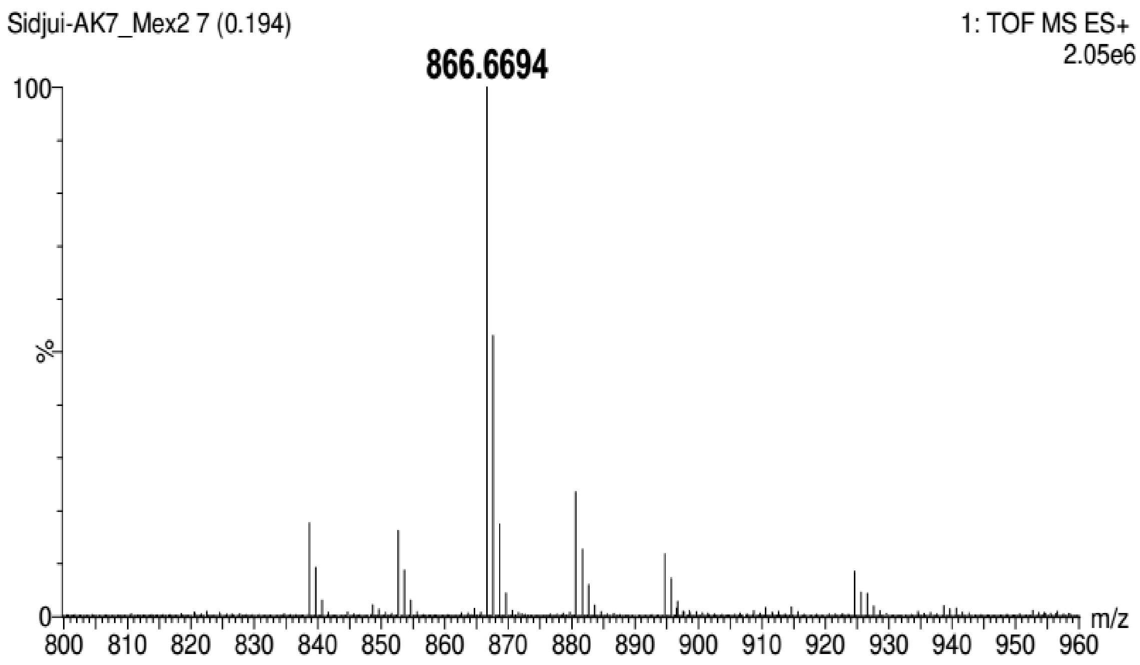

Figure S4: HR-ESI-MS (+) of compound **1**

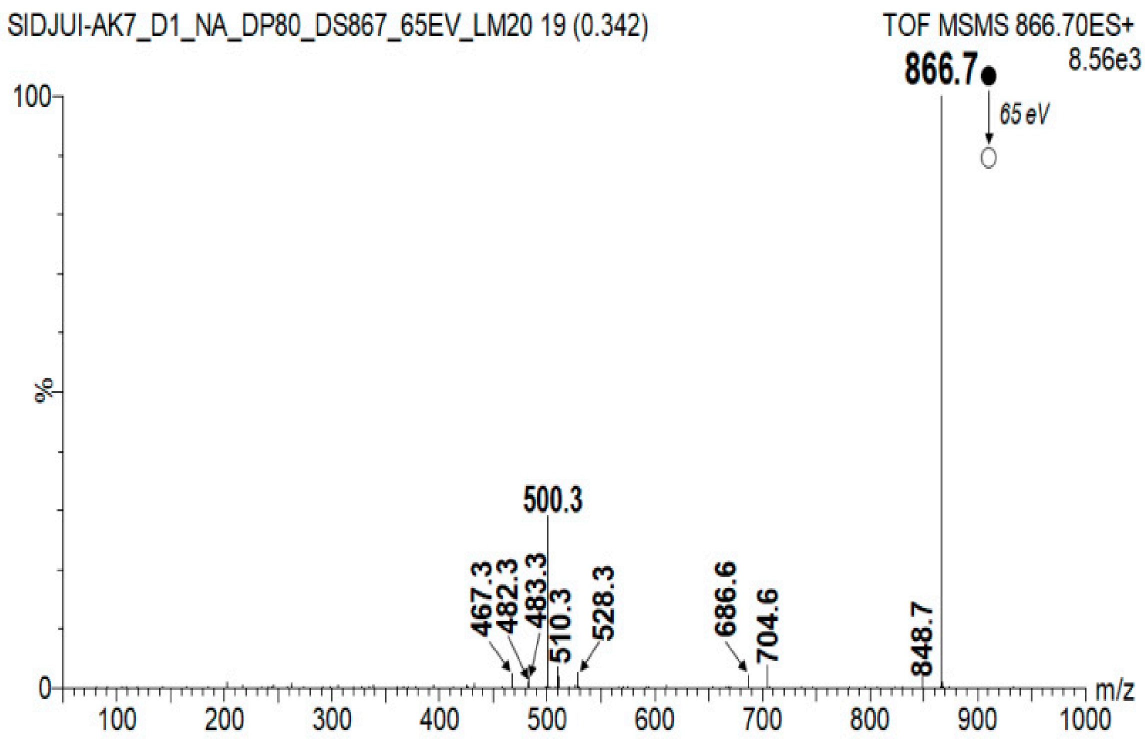

Figure S5: MS/MS spectrum of the $[M+Na]^+$ ion at *m/z* 866.7 (Ecollision = 65 eV) of compound **1**

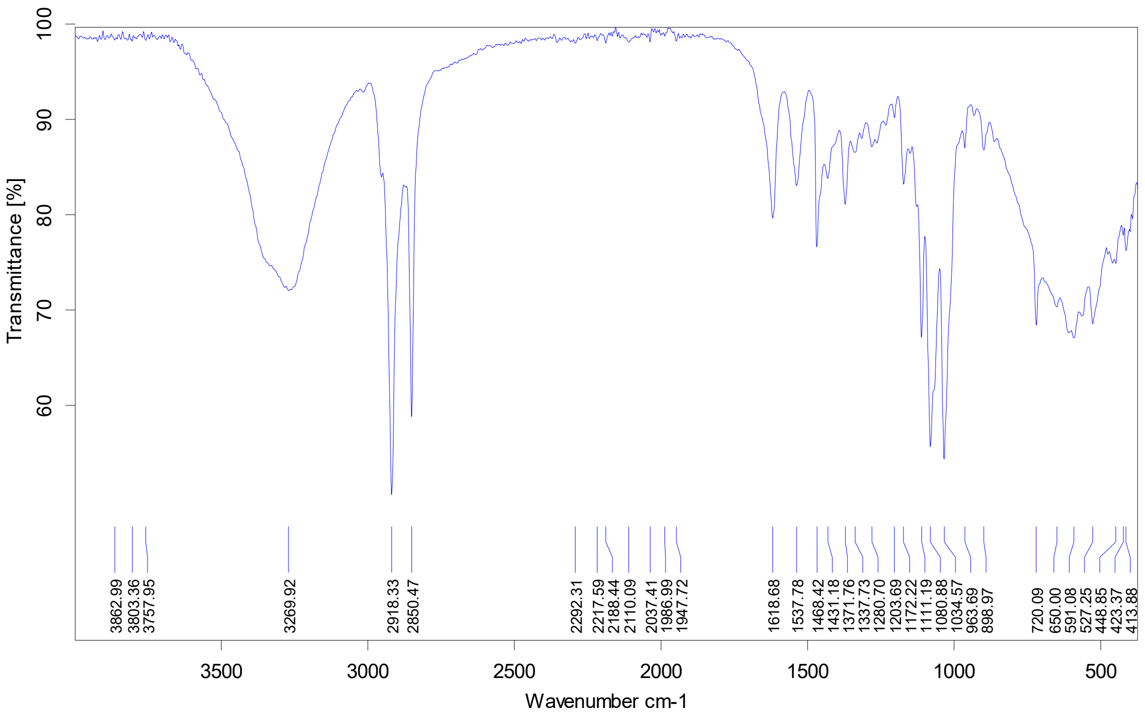

Figure S6: IR spectrum (KBr) of compound **1**

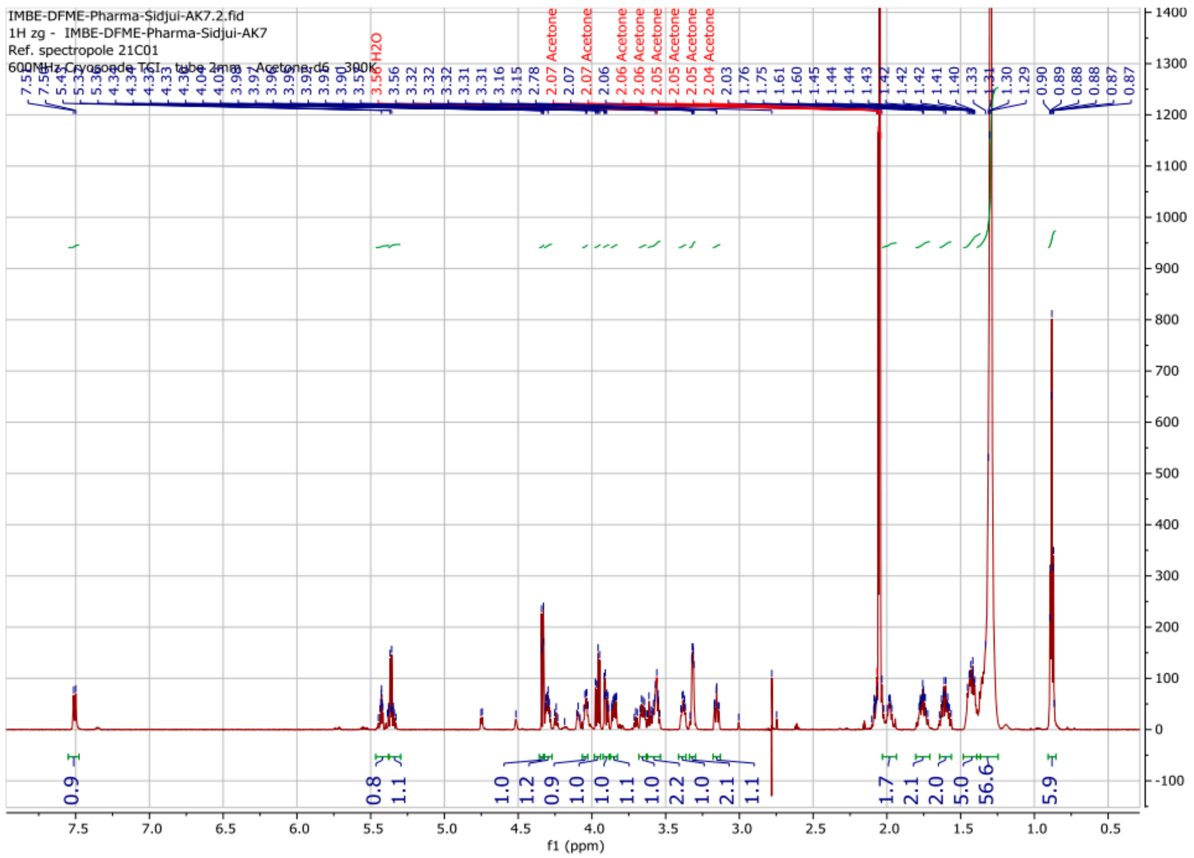

Figure S7: ^{1}H-NMR (Acetone-d_6, 600 MHz) spectrum of compound **1**

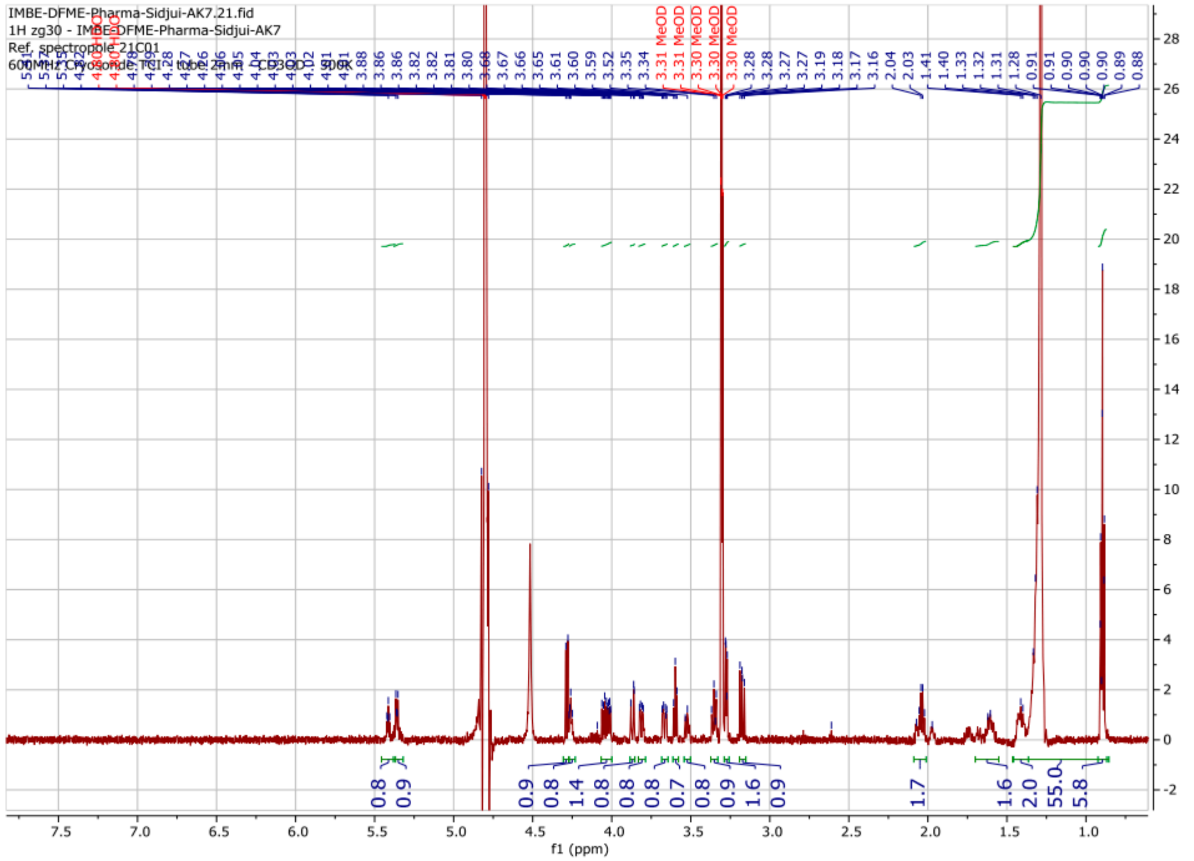

Figure S8: ^{1}H-NMR (CD_3OD, 600 MHz) spectrum of compound **1**

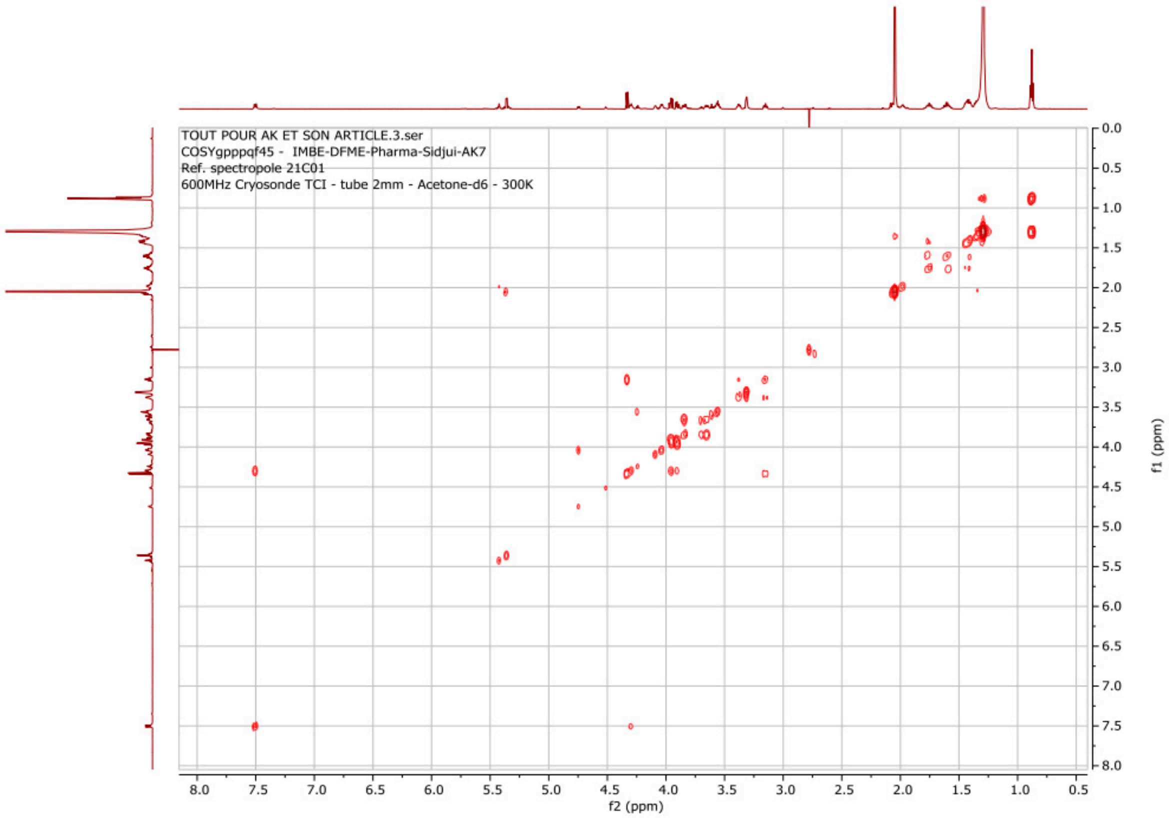

Figure S9: COSY (Acetone-d_6) full spectrum of compound **1**

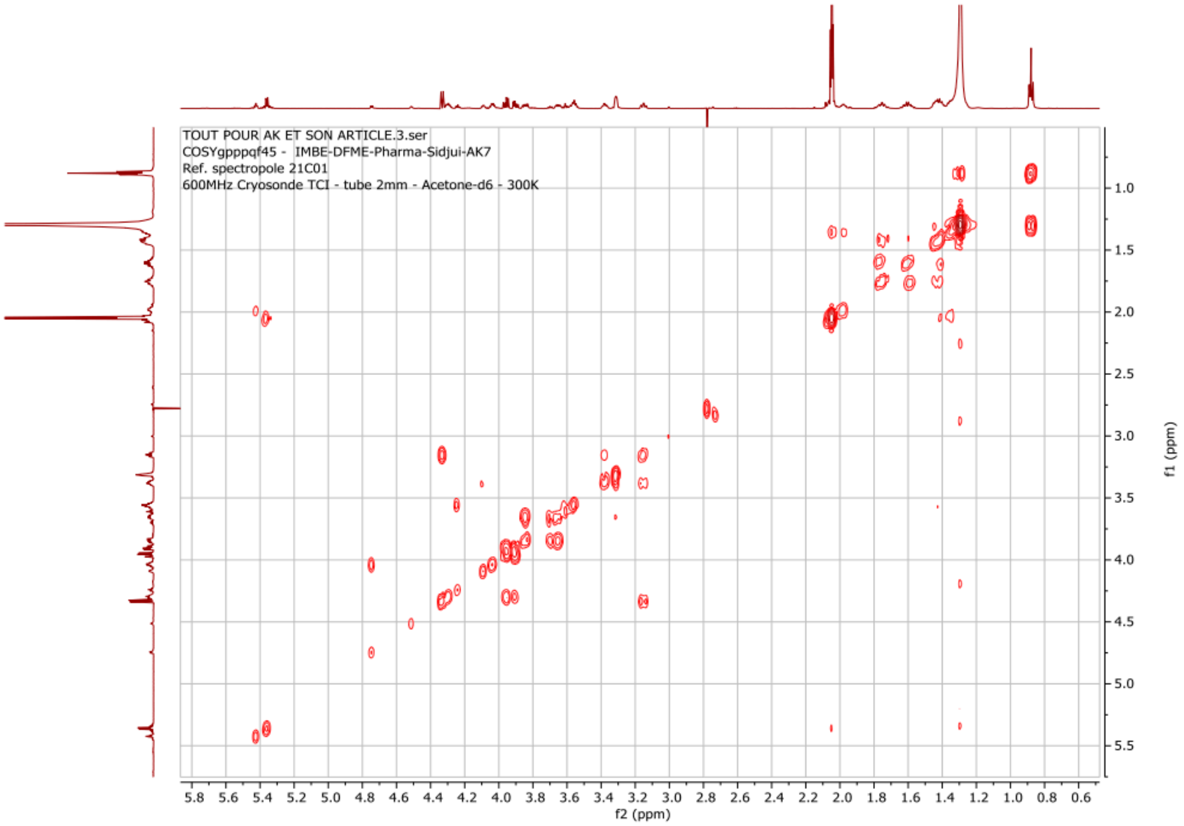

Figure S10: COSY (Acetone-d_6) expanded spectrum of compound **1**

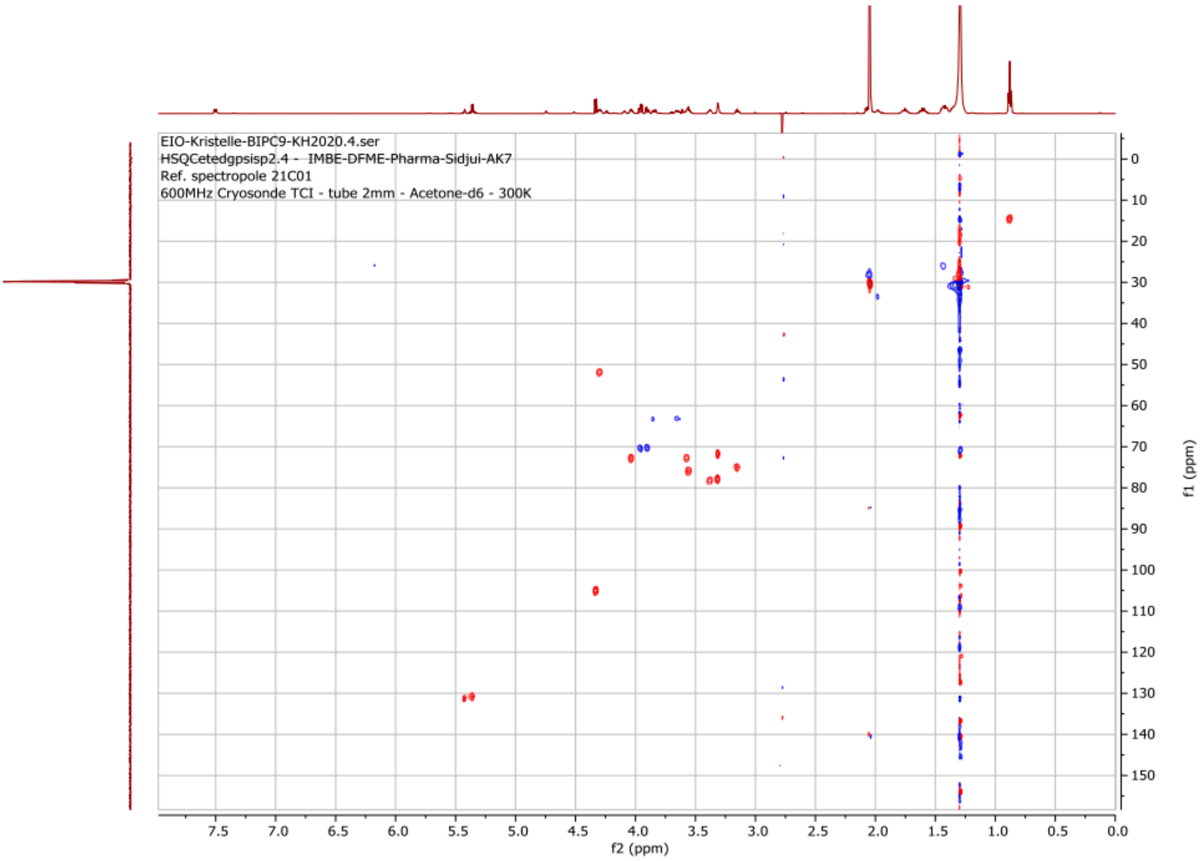

Figure S11: HSQC (Acetone-d_6) spectrum of compound **1**

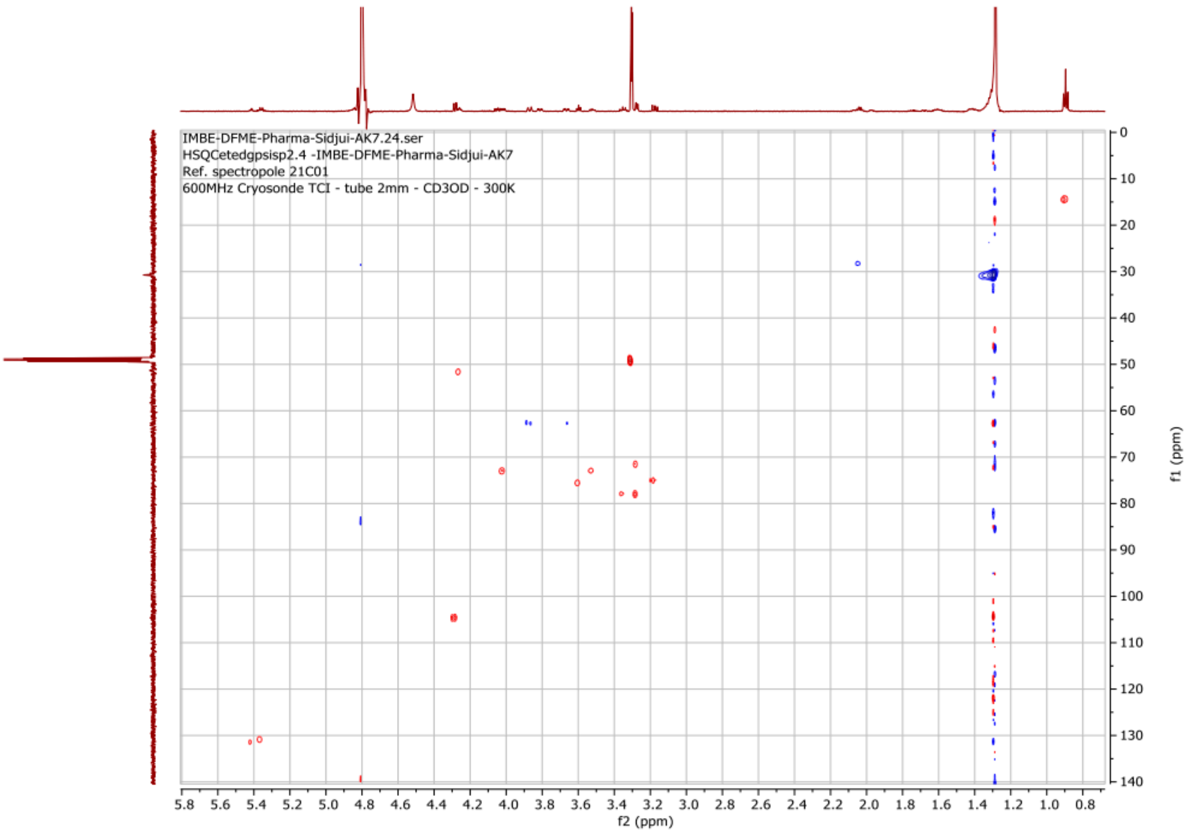

Figure S12: HSQC (CD_3OD) spectrum of compound **1**

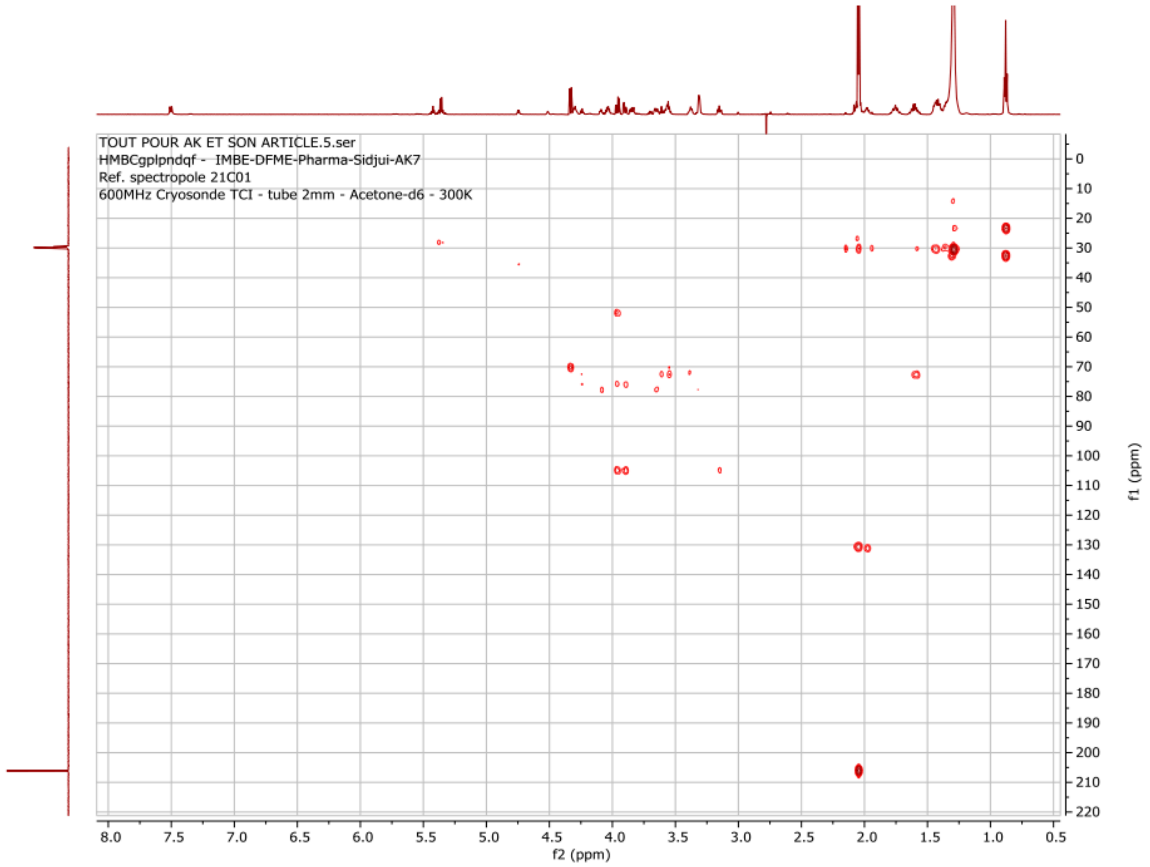

Figure S13: HMBC (Acetone-d_6) spectrum of compound **1**

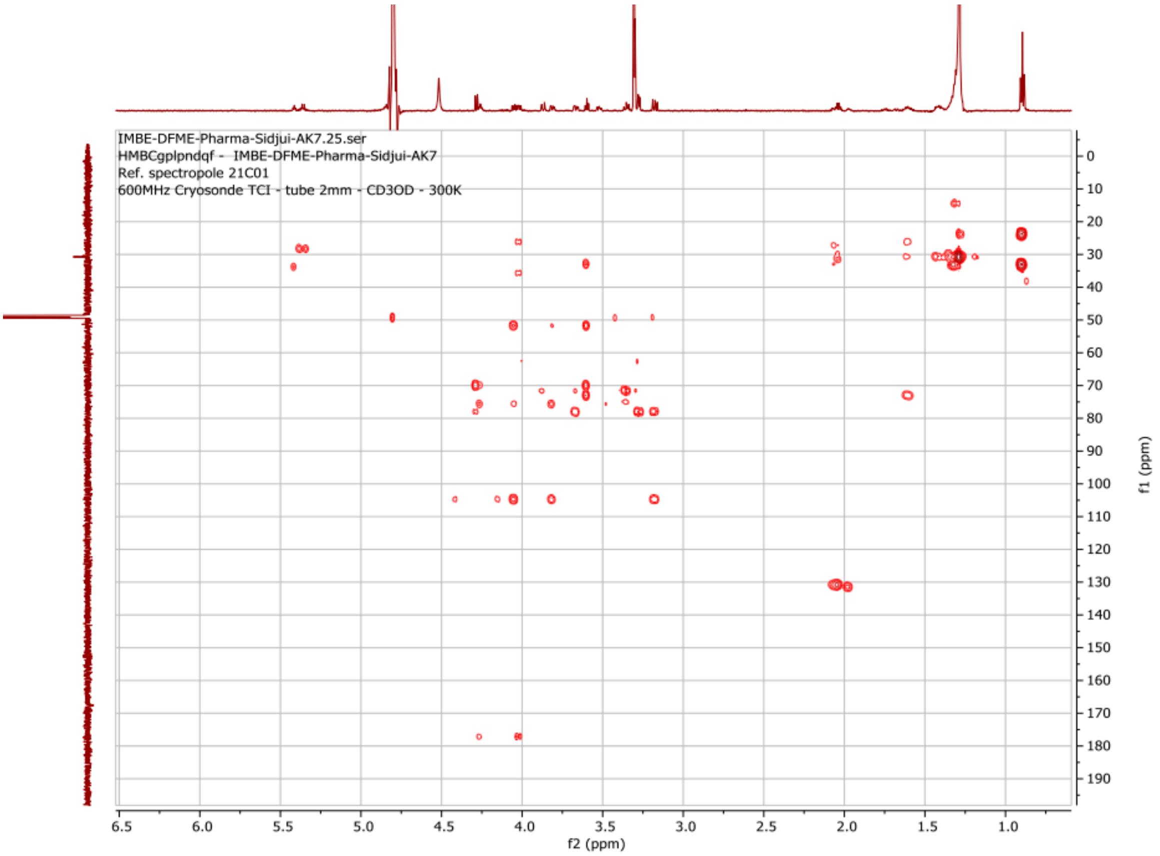

Figure S14: HMBC (CD_3OD) spectrum of compound **1**

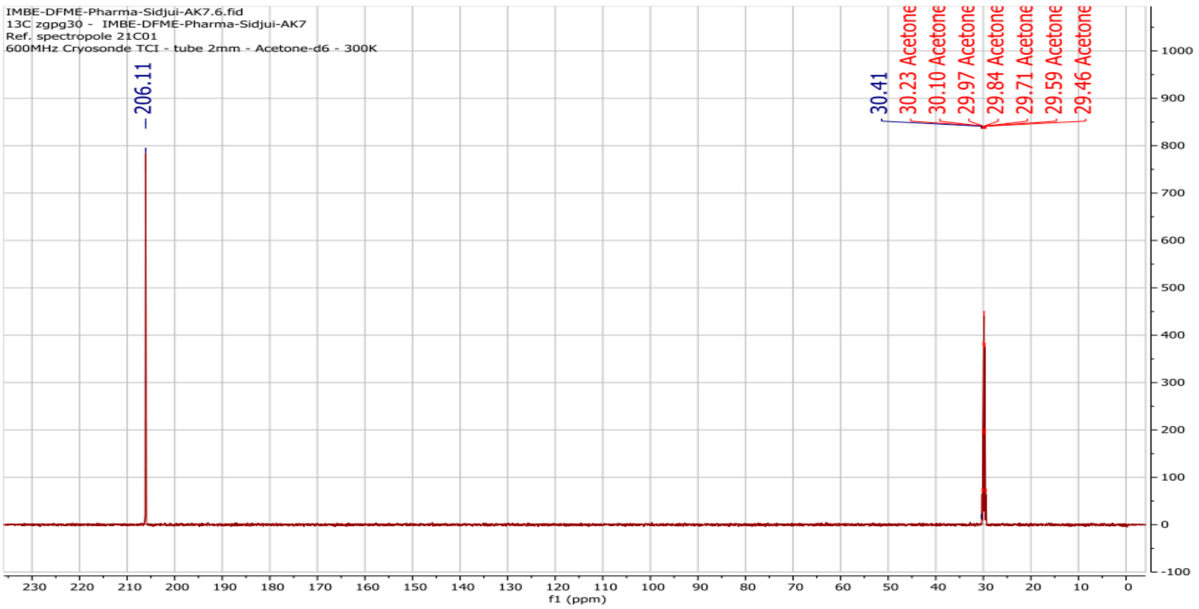

Figure S15: ^{13}C-NMR (Acetone-*d*$_6$, 150 MHz) spectrum of compound **1**

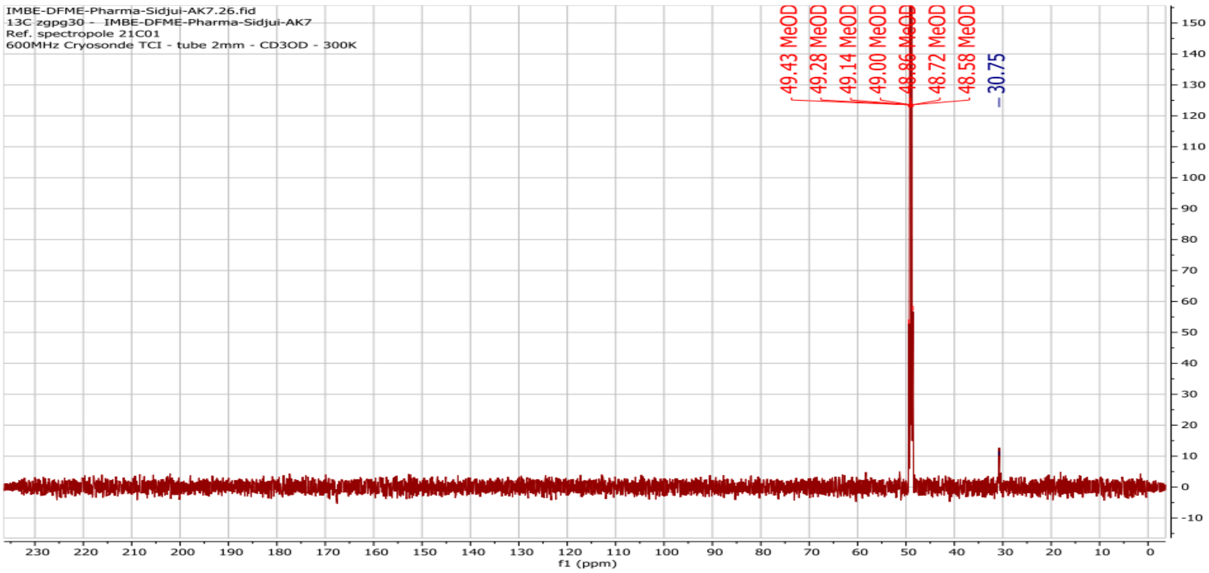

Figure S16: ^{13}C-NMR (CD_3OD, 150 MHz) spectrum of compound **1**

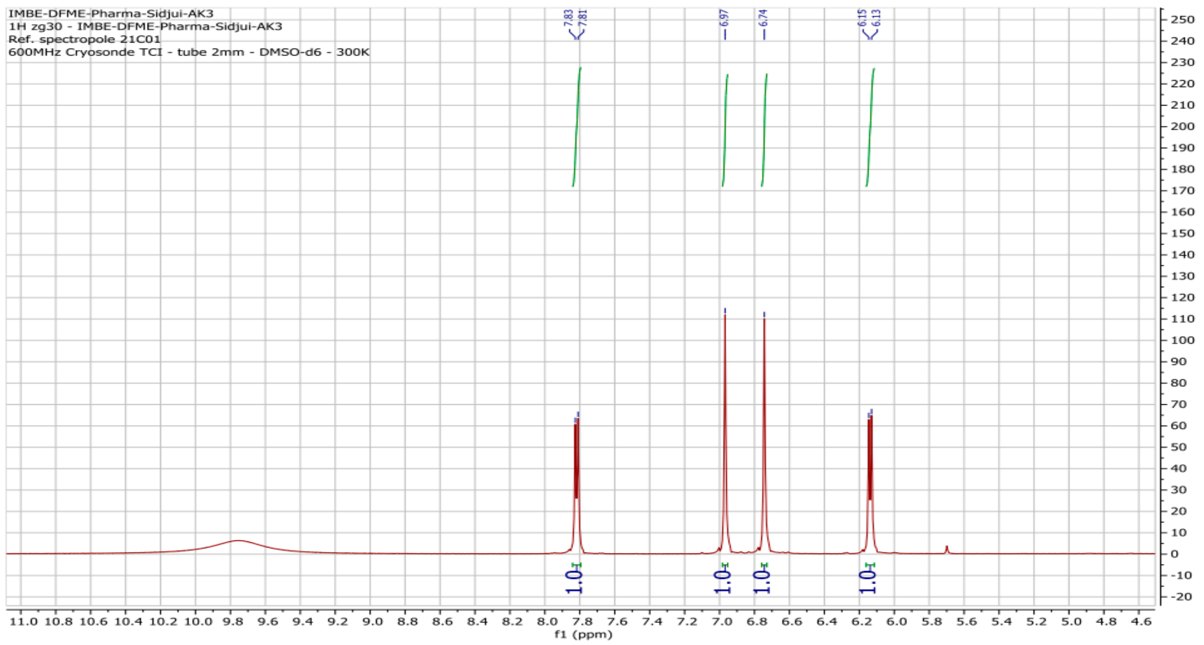

Figure S17: ^{1}H-NMR (DMSO-d_6, 600 MHz) spectrum of compound **2**

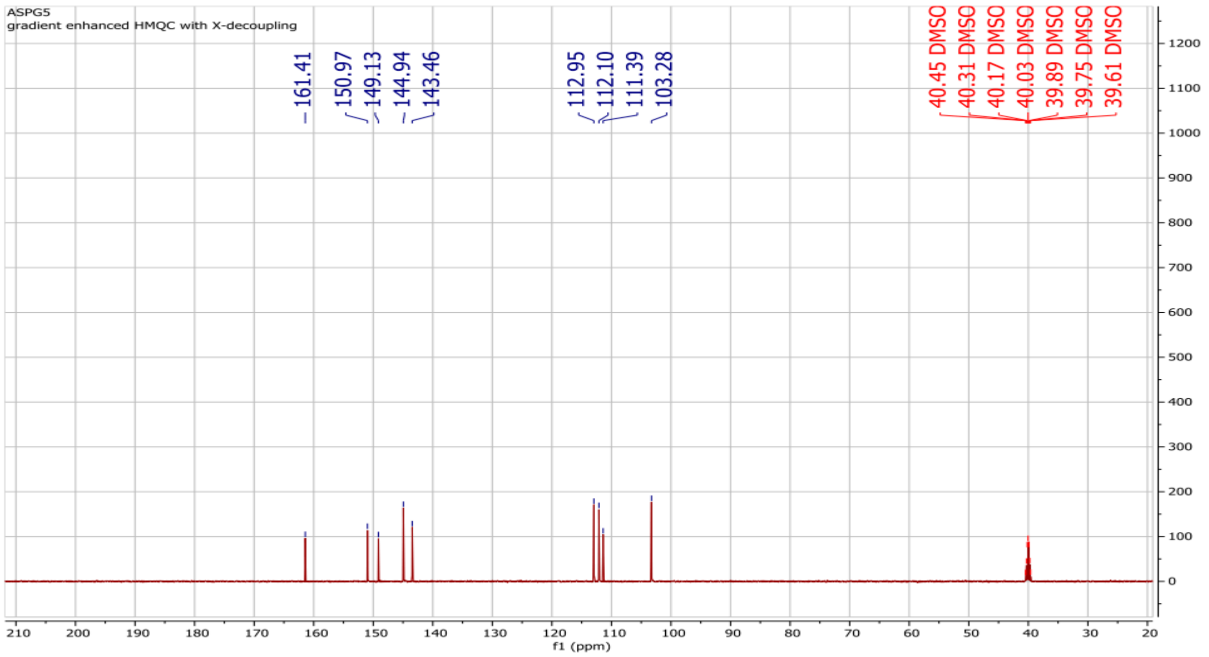

Figure S18: ^{13}C-NMR (DMSO-d_6, 150 MHz) spectrum of compound **2**

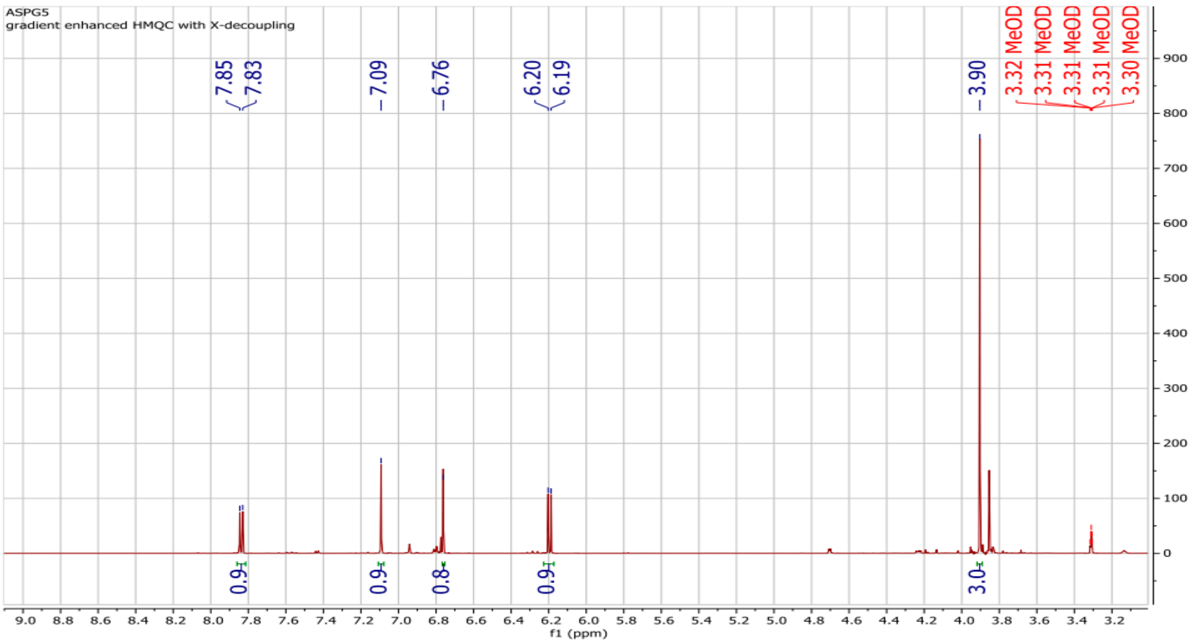

Figure S19: ^{1}H-NMR (CD_3OD, 600 MHz) spectrum of compound **3**

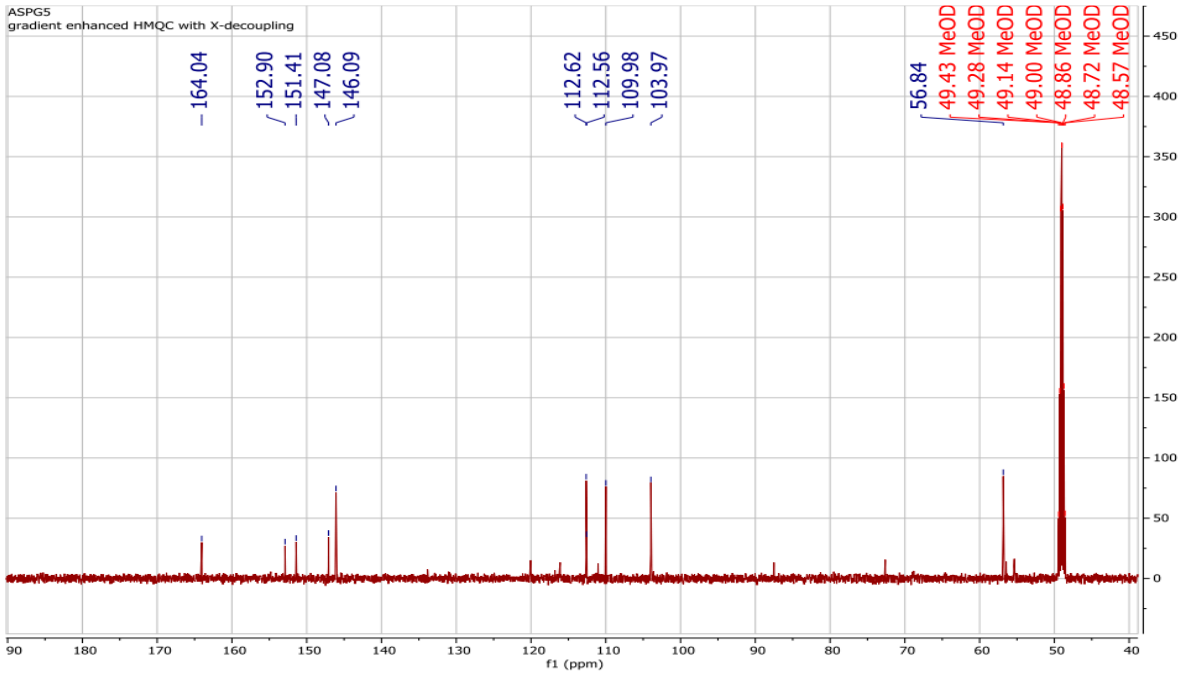

Figure S20: ^{13}C-NMR (CD_3OD, 150 MHz) spectrum of compound **3**

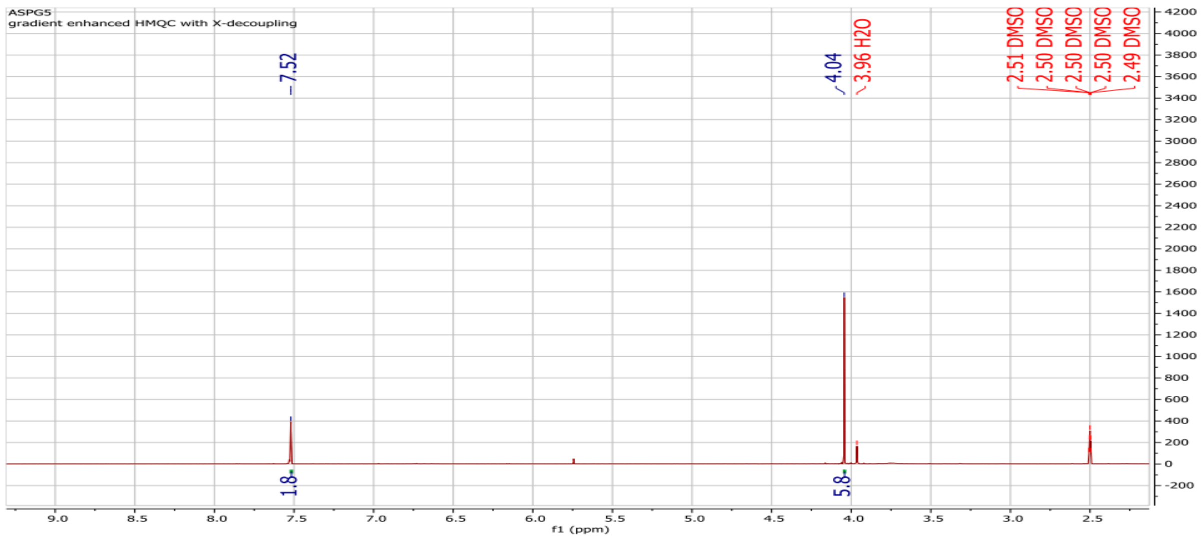

Figure S21: ^{1}H-NMR (DMSO-d_6, 600 MHz) spectrum of compound **4**

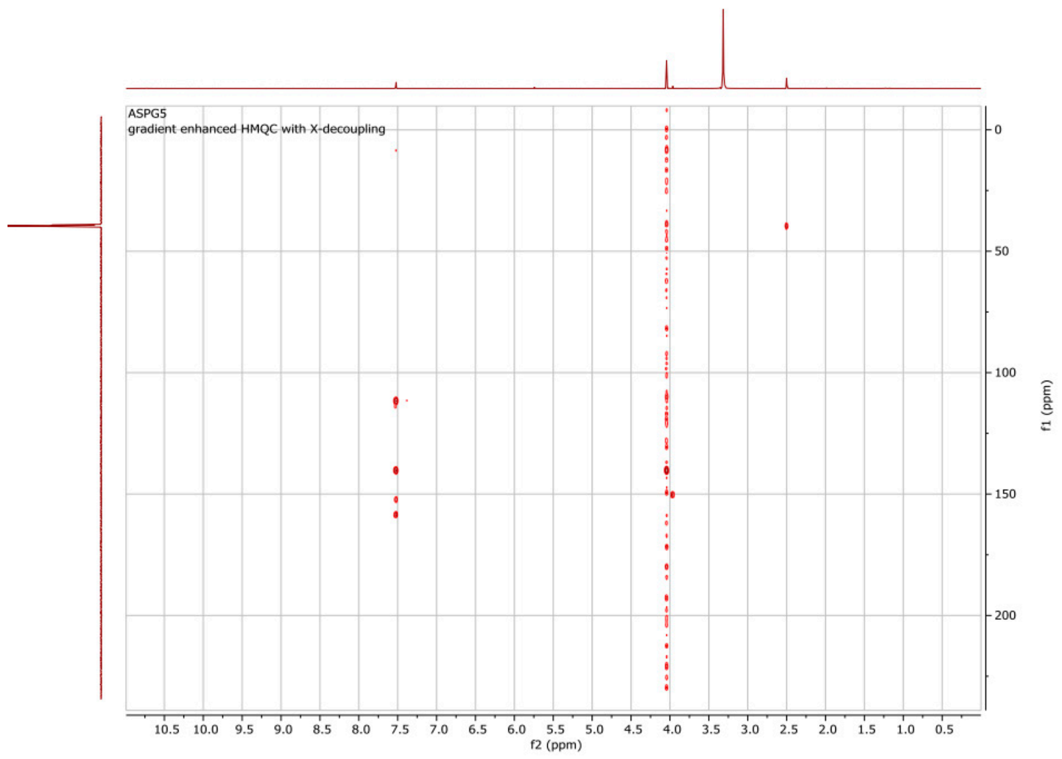

Figure S22: HMBC spectrum of compound **4**

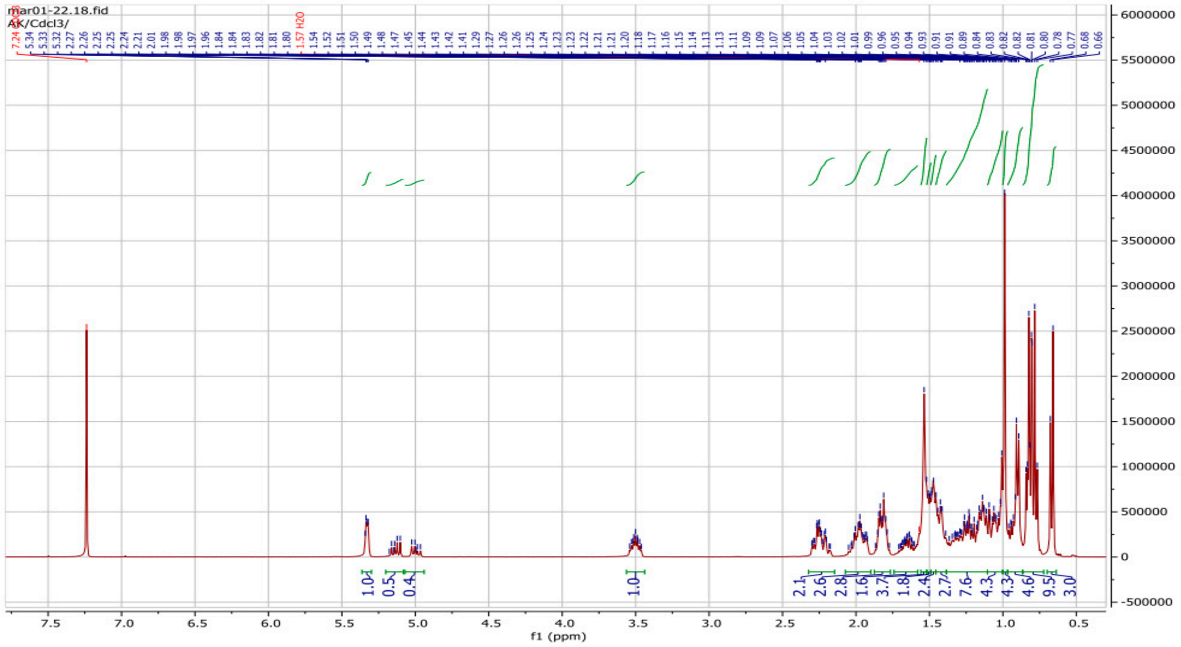

Figure S23: ^{1}H-NMR ($CDCl_3$, 600 MHz) spectrum of compound **5 & 6**
